# Mathematical modeling and optimization technique of anticancer antibiotic adsorption onto carbon nanocarriers

**DOI:** 10.1038/s41598-024-62483-4

**Published:** 2024-05-25

**Authors:** Kanes Sumetpipat, Duangkamon Baowan, Prangsai Tiangtrong

**Affiliations:** 1 Department of Mathematics and Computer Science, Kamnoetvidya Science Academy, Rayong, 21210 Thailand; 2 Department of Mathematics, Faculty of Science, Mahidol University, Rama VI Rd., Bangkok, 10400 Thailand; 3 Department of Mathematics, Faculty of Science, Ramkhamhaeng University, Ramkhamhaeng Rd., Bangkok, 10240 Thailand

**Keywords:** Adsorption energy, U-NSGA-III algorithm, Lennard–Jones potential, Carbon, Fluorouracil, Proflavine, Methylene blue, Graphene, Nanoscale materials, Applied mathematics, Computer science, Theory and computation

## Abstract

This study employs a combination of mathematical derivation and optimization technique to investigate the adsorption of drug molecules on nanocarriers. Specifically, the chemotherapy drugs, fluorouracil, proflavine, and methylene blue, are non-covalently bonded with either a flat graphene sheet or a spherical ${\rm C}_{60}$ fullerene. Mathematical expressions for the interaction energy between an atom and graphene, as well as between an atom and ${\rm C}_{60}$ fullerene, are derived. Subsequently, a discrete summation is evaluated for all atoms on the drug molecule utilizing the U-NSGA-III algorithm. The stable configurations’ three-dimensional architectures are presented, accompanied by numerical values for crucial parameters. The results indicate that the nanocarrier’s structure effectively accommodates the atoms on the drug’s carbon planes. The three drug types’ molecules disperse across the graphene surface, whereas only fluorouracil spreads on the ${\rm C}_{60}$ surface; proflavine and methylene blue stack vertically to form a layer. Furthermore, all atomic positions of equilibrium configurations for all systems are obtained. This hybrid method, integrating analytical expressions and an optimization process, significantly reduces computational time, representing an initial step in studying the binding of drug molecules on nanocarriers.

## Introduction

Developing novel techniques for drug delivery to target cells is crucial, representing a formidable challenge in pharmacological innovation. A promising avenue in this pursuit involves the utilization of specific nanoscaled carriers. The application of these delivery platforms is designed to achieve several critical objectives, including enhanced precision in drug targeting, reduced toxicity to the body, heightened safety and bio-compatibility, secure development of new medications, avoidance of effects stemming from medication resistance mechanisms, and improved rates and quantities of medicine absorption^1–4^.

A number of studies investigate the mechanics of nanocarriers using various methods to facilitate comprehensive analysis. Ibrahim et al.^5^ utilize the density functional theory method (DFT) to explore the adsorption of the anti-Covid-19 drug Favipiravir (FPV) on the fullerene-like nanocarrier Al12N12, an aluminum nitride nanocarrier. Similarly, Bibi et al.^6^ employ DFT to analyze the potential of three metal-doped fullerenes, CrC_59_, FeC_59_, and NiC_59_ as carriers for FPV. Their findings suggest that these nanocarriers hold promise in enhancing delivery rates, minimizing non-specific accumulation, mitigating adverse effects, and regulating drug release at the target location.

Sun et al.^7^ determine the interaction between graphene and enzyme for the design of artificial inhibitors where they investigate the absorption of $\alpha$-chymotrypsin (ChT) on pristine graphene and that on graphene oxide. They further undertake the molecular dynamics simulations to calculate the docking scores of proflavine docking into ChT, with and without pristine graphene. Yaraghi et al.^8^ observe the energies and atomic properties of tautomers in fluorouracil with silicon graphene nanosheet using DFT. Their findings reveal that the model of di-keto fluorouracil shows the lowest interaction energy and is the most stable among the tautomers considered. Moreover, Neal et al.^9^ employ DFT to optimize the interactions between ${\rm C}_{60}$ and doxorubicin (DOX), epirubicin, and mitomycin. They report their findings regarding the non-covalent interaction energies and configurations in which the drug rings are parallel to the five or six-membered rings of ${\rm C}_{60}$.

Evstigneev et al.^10^ investigate the complexation of ${\rm C}_{60}$ in aqueous solution with DOX, methylene blue and proflavine via atomic force microscopy to provide a potential application in biotechnology. Sabet et al.^11^ theoretical assessment of the solvent effect on functionalization of Au_32_ and ${\rm C}_{60}$ nanocages with fluorouracil. DFT calculations are used to simulate the interaction of fluorouracil with Au_32_ and ${\rm C}_{60}$ fullerenes. The results show that fluorouracil binds more strongly to Au_32_ than C_60_. However, in water, fluorouracil is not able to bind to either Au_32_ or C_60_.

Furthermore, several studies have successfully derived explicit formulas for the interaction energies between drug molecules and nanocarriers, which may be utilized to determine the conditions for acceptance and encapsulation behaviors for various medicines and nanoparticles. Alshehri^12^ investigates the encapsulation of three anticancer medicines, namely cisplatin, carboplatin, and DOX, within three distinct kinds of nanotubes which are carbon, silicon, and boron nitride nanotubes. He employs the Lennard-Jones potential function together with the integration technique to derive the energy function in terms of nanotube radii for individual drug. With the similar technique, Putthikorn et al.^13^ investigate the interaction energy between a hollow sphere of DOX and the cyclo[(-D-Ala-L-Ala)_4_-] peptide nanotube. The findings of their study demonstrate the optimal radius of DOX that can be encapsulated in the cyclic peptide nanotube and the drug molecule is expected to be situated within the gap regions among the sub-units of the cyclic peptide nanotube. Indeed, numerous investigations have been conducted utilizing elementary mechanics and mathematical models to derive specific formulae for the interaction energy between different chemical compounds^14–19^.

In this paper, we use mathematical models to calculate the interaction energy between an atom and a nanocarrier. The atom is assumed to be on the drug molecule, and an optimization technique is then applied to evaluate the total energy of the system. We focus on three types of drug molecules: fluorouracil (C_4_H_3_FN_2_O_2_) a straightforward treatment for various cancer types; proflavine (C_13_H_11_N_3_) studied as an intercalator in cancer treatments and RNA-targeted antiviral medications^20^; and methylene blue (C_16_H_18_ClN_3_S) an FDA-approved dye and drug for treating methemoglobinemia. Due to their planar structure, we aim to investigate the relative distances of these three drug molecules from the carrier. Explicit formulas for each system are employed to find the minimum energy between drugs and two carbon-based material carriers which are graphene sheet and C_60_ fullerene. The Unified Non-dominated Sorting Genetic Algorithm III (U-NSGA-III), an evolutionary algorithm for optimization, assesses the stability of the systems. This hybrid method successfully used by the authors in a previous work^21^ significantly reduces computational calculation time.

The paper is structured as follows: the next section presents mathematical derivations for van der Waals energy and optimization formulations. Section 3 provides detailed parameter descriptions and nomenclature definitions, along with the presentation of numerical findings for the studied systems. Section 4 presents a comparison between our results and other works. A summary is made in Sect. 5. Energy value comparisons from continuous, discrete, and hybrid approaches, an example of the objective function for the optimization process, and drug molecule alignments are detailed in Supplementary Material S1.

## Interaction energy and model formation

We employ the Lennard-Jones potential function to calculate the molecular interatomic energy for two non-bonded atoms which can be written as

1 $$\begin{aligned} \Phi = -\frac{A}{\rho ^6} + \frac{B}{\rho ^{12}} = \epsilon \left[ -2\left( \frac{\sigma }{\rho }\right) ^6 + \left( \frac{\sigma }{\rho }\right) ^{12} \right] , \end{aligned}$$

where $\rho$ denotes the distance between two typical points, and $A=2\epsilon \sigma ^6$ and $B=\epsilon \sigma ^{12}$ are attractive and repulsive Lennard-Jones constants, respectively. Further, $\epsilon$ denotes a well depth and $\sigma$ is the van der Waals diameter. Moreover, the mixing rule is utilized in the system of two atomic species which are $\epsilon _{ij} = \sqrt{\epsilon _i\epsilon _j}$ and $\sigma _{ij}=(\sigma _i+\sigma _j)/2$. The Lennard-Jones parameters for atoms used in this study are taken from the work of Rappe et al.^22^.

For two non-bonded molecular structures, the interaction energy can be evaluated using either a discrete atom-atom formulation or by a continuous approach. In the interest of modeling irregularly shaped molecules, such as drugs, an alternative hybrid discrete-continuous approximation can also be used which is given by

$$\begin{aligned} E^{tot}= \eta \sum _{i}{\int \Phi \left( \rho _i\right) }dS, \end{aligned}$$

where $\eta$ is the surface density of atoms on the molecule which is considered continuous, $\rho _i$ is the distance between a typical surface element *dS* on the continuously modeled molecule and atom *i* in the molecule which is modeled as discrete. The total energy is obtained by discretely summing over all atoms in the drug.

The continuous approach is assumed that the atoms at discrete locations on the molecule are averaged over a surface and the molecular interatomic energy is obtained by calculating integrals over the surface of the molecule. On using the Lennard-Jones function defined by (1), the interaction energy between a point (single atom) and a carrier is given by

$$\begin{aligned} E_{p} = \eta \int _{S} \left( -\frac{A}{\rho ^6} + \frac{B}{\rho ^{12}}\right) dS. \end{aligned}$$

For convenience, we define

2 $$\begin{aligned} I_n = \int _{S} \frac{1}{\rho ^{2n}}dS, \qquad n = 3, 6, \end{aligned}$$

and $E_p=\eta (-AI_3 + BI_6)$. The energy comparison arises from the continuous, discrete and hybrid models are given in Supplementary Material S1.

### Interaction between point and plane

**Figure 1:**
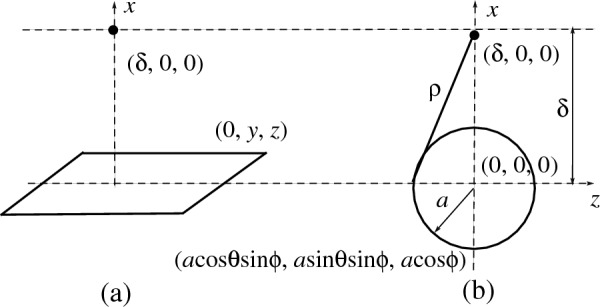
Schematic models for interaction between point and surfaces of (**a**) infinite flat plane and (**b**) sphere of radius *a*.

First, we consider the interaction energy between a point *P* located at $(\delta ,0,0)$ and an infinite plane (0, *y*, *z*) as shown in Fig. 1a. The distance between the point *P* to the plane is $\rho _p = \sqrt{\delta ^2+y^2+z^2}$ and the integral $I_n$ becomes

$$\begin{aligned} I_n^p = \int _{-\infty }^{\infty } \int _{-\infty }^{\infty } \frac{1}{(\delta ^2+y^2+z^2)^n} dxdy. \end{aligned}$$

On using the substitution and changing the limit of integration (see^23^ for the integration details), we may deduce $I_n^p = \frac{\pi }{(n-1)\delta ^{2n-2}}$. Hence, the interaction energy between a point and the infinite plane is given by

3 $$\begin{aligned} E_{pp} = \eta _p\pi \left( -\frac{A}{2\delta ^4} + \frac{B}{5\delta ^{10}}\right) , \end{aligned}$$

where $\eta _p$ is the mean atomic surface density of the graphene and it is given by 0.3812 atom/Å^2^, and $\delta$ is the distance between each atom on the drug and the graphene. Note that a graphene sheet consists of a tessellation of hexagonal rings, so that each atom contributes one-third of the total number of atoms in the ring. Since there are six carbon atoms per hexagon, the surface density of carbon atoms is thus

$$\begin{aligned} \eta _p = \frac{1/3 \times 6}{3\sqrt{3}\sigma ^2/2} = \frac{4}{3\sqrt{3}\sigma ^2} = 0.3812, \end{aligned}$$

where $\sigma = 1.42$ Å which is a carbon-carbon bond length in graphene.

### Interaction between point and sphere

We consider the interaction of a spherical surface of radius *a* centered at the origin and a point located at the $(\delta ,0,0)$ as shown in Fig. 1b. The distance from a typical element on the surface of the sphere to the atom is given by $\rho ^2_s = (a\cos \theta \sin \phi )^2 +(a\sin \theta \sin \phi )^2 +(a\cos \phi - \delta )^2.$ The integral $I_n$ defined by (2) becomes

$$\begin{aligned} I_n^s = \int _{-\pi }^{\pi } \int _0^{\pi } \frac{a^2\sin \phi }{(a^2+\delta ^2-2a\delta \cos \phi )^n} d\phi d\theta . \end{aligned}$$

By making the substitution $t=a^2+\delta ^2-2a\delta \cos \phi$ and using the fact that $I_n^s$ is independent of $\theta$, we deduce

4 $$\begin{aligned} I_n^s = \frac{a\pi }{\delta } \int _{(\delta -a)^2}^{(\delta +a)^2} \frac{1}{t^n} dt = \frac{a\pi }{\delta (n-1)}\left[ \frac{1}{(a-\delta )^{2(n-1)}} - \frac{1}{(a+\delta )^{2(n-1)}} \right] . \end{aligned}$$

Therefore, the total interaction energy between a surface of a sphere and a single atom is given by

5 $$\begin{aligned} E_{sp} = \frac{a\pi \eta _s}{\delta }\left\{ -\frac{A}{2}\left[ \frac{1}{(a-\delta )^4}-\frac{1}{(a+\delta )^4} \right] +\frac{B}{5}\left[ \frac{1}{(a-\delta )^{10}}-\frac{1}{(a+\delta )^{10}} \right] \right\} , \end{aligned}$$

where $\eta _s = 60/4\pi a^2 = 0.3789$ atom/Å^2^ is the mean atomic surface density of the C_60_ fullerene of radius $a=3.55$ Å, and $\delta$ is the distance from the spherical center to the point.

### Problem formulations and optimization process

The coordinates of a C_60_ are taken from CHEMICAL COMPOUNDS DEEP DATA SOURCE (CCDDS) (https://www.molinstincts.com/sdf-mol-file/Fullerene-C60-sdf-CT1002894513.html), and the coordinates of fluorouracil, proflavine, and methylene blue molecules are obtained from PubChem database of National Institutes of Health (NIH) with PubChem CID 3385, 7099 and 6099, respectively^24^.

All molecules are treated as rigid with no bending or twisting, and the default position and conformation are set at the origin (0, 0, 0). For molecular manipulation, we explore new positions and conformations by adjusting five variables $(x, y, z, \theta _x, \theta _y)$. This means the molecule can be relocated to any position (*x*, *y*, *z*) and rotated by angles $\theta _x$ and $\theta _y$ along the *x*-axis and *y*-axis, respectively. This leads to a total of 5*n* decision variables for *n* molecule(s), where *n* is the number of molecules in a system. During computation, *x*, *y*,  and *z* are restricted to the range of $-16$ to 16 Å, and $\theta _x$ and $\theta _y$ are confined to the range of $-180^{\circ }$ to $180^{\circ }$. However, the graphene sheet is handled differently, assumed to be an ideal infinite flat plane located on the *xy*-plane.

Optimization techniques play a significant role in current academic research and industry challenges. The Non-dominated Sorting Genetic Algorithm (NSGA) is a robust algorithm in both artificial intelligence and optimization methods, particularly effective in solving multi-objective problems. NSGA-III^25^ has demonstrated high performance in finding optimal solutions for multiple objective problems. Similarly, the Unified NSGA-III (U-NSGA-III) has proven effective in addressing various practical problems when adapted to a single objective^26^. The steps of a genetic algorithm to find the optimal solution are as follows

1. Initialize parameters such as population size and the maximum number of generations.
2. Each individual in the initial population represents a potential solution to the optimization problem addressed in that generation.
3. Produce each subsequent population from the parents of the previous generation through genetic operations like crossover and mutation. Consequently, each new population typically comprises solutions that, on average, outperform their parental generation. Over successive generations, the quality of solutions tends to improve.
4. Upon reaching the final generation, evaluate and select the best population among all populations generated throughout the algorithm’s execution. This optimal population represents either the optimal or near-optimal solutions to the optimization problem.

In this study, the U-NSGA-III algorithm from the pymoo library is utilized^27^, following default choices for sampling, tournament selection, crossover, and mutation processes. Some parameters, such as pop_size = $12{,}000-15{,}000$, n_offsprings = 8000–12,000, and n_gen $=400-600$, are manually set. Note that n_gen is increased beyond 400 when optimizing the interaction energy of multiple drug molecules. Each optimization problem is run 5–12 times with different seed numbers. The minimum energy from the objective function and its optimal location from each seed are recorded (see Supplementary Material S1 for an example of the energy objective function). Then all optimal energy values are compared and grouped as different configurations. Additional details on problem formulations and optimization settings can be found in Sumetpipat and Baowan^21^.

## Numerical results

In this section, we define key parameters to characterize molecular alignments. The results of numerical experiments regarding the interaction energy between drug molecules and nanocarriers are presented and interpreted.

### Parameter descriptions

**Figure 2:**
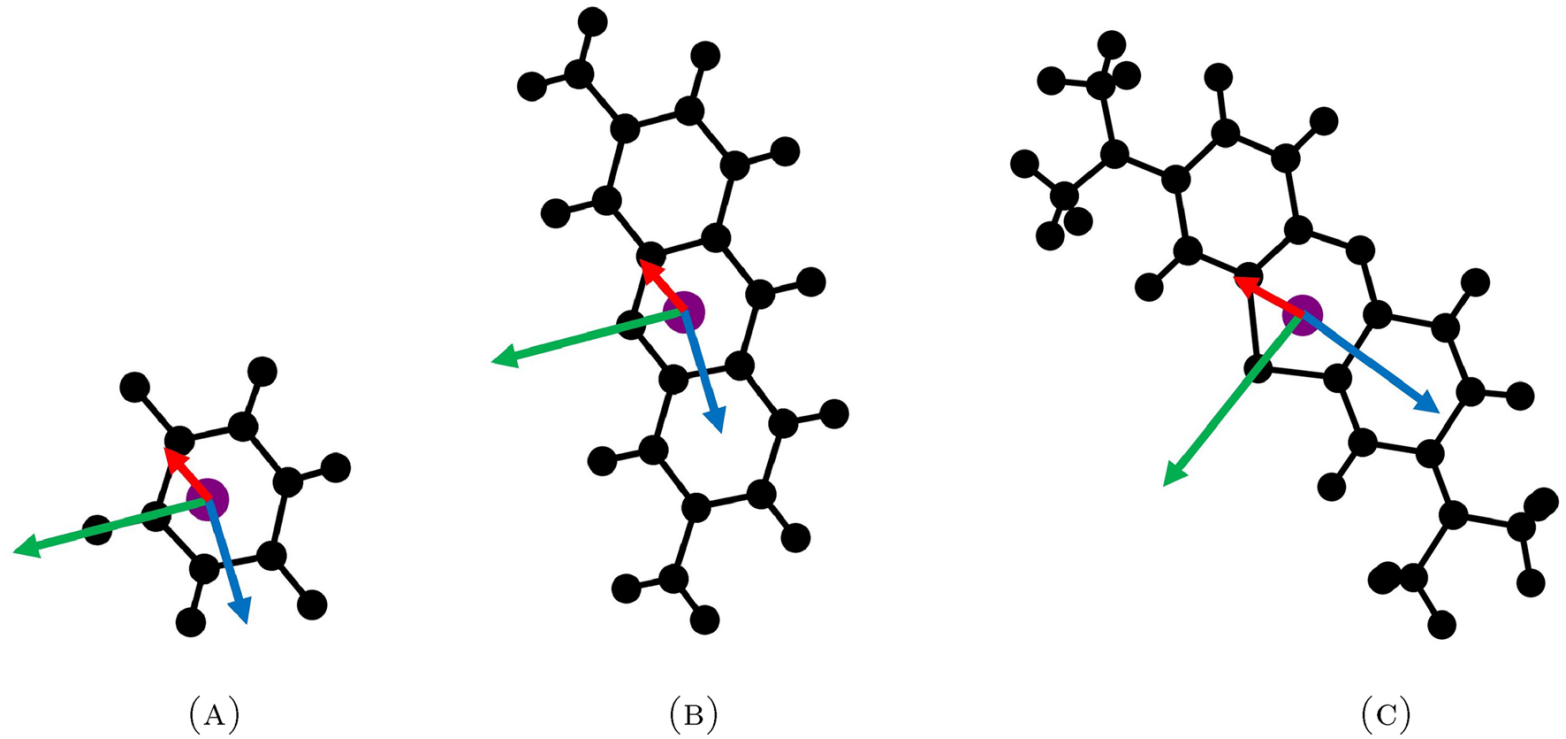
Vector representations for a molecule of (**A**) fluorouracil, (**B**) proflavine and (**C**) methylene blue.

To provide numerical insights into how molecules form and their relative positions at equilibrium, we create three vectors which are front vector, side vector, and normal vector for each drug type, as shown in Fig. 2. Initially, we determine the molecular center of mass, represented by the purple circle. Then, we draw the green front vector from the center of mass to the fluoride, oxygen, and nitrogen atoms on fluorouracil, proflavine, and methylene blue, respectively. The blue side vector is parallel to the carbon plane, and the red normal vector is orthogonal to the carbon plane. We note that methylene blue is an oxidation-reduction agent and it is reversible between methylene blue and leucomethylene blue (colourless). The chloride ion itself does not directly participate in the properties of methylene blue, it is related to its use as a dye or medication. In this calculation, the chloride is not taken into account to the energy contribution and the molecular structure of the methylene blue is depicted in Fig. 2c.

In terms of the alignment, these three vectors serve as a representation, allowing us to measure how drug molecules align by assessing the angle between their carbon planes. This representation also helps us understand how drug molecules interact with the surface of a nanocarrier. It is important to note that due to asymmetry in at least one dimension and the non-planar structure of methylene blue, two drug molecules are not perfectly parallel. Therefore, our focus is on examining the positioning of molecules that can be parallel and the front vectors point in the same direction (co-parallel), parallel but the front vectors point in the opposite direction (anti-parallel) or tilted parallel (orthogonally parallel).

**Figure 3:**
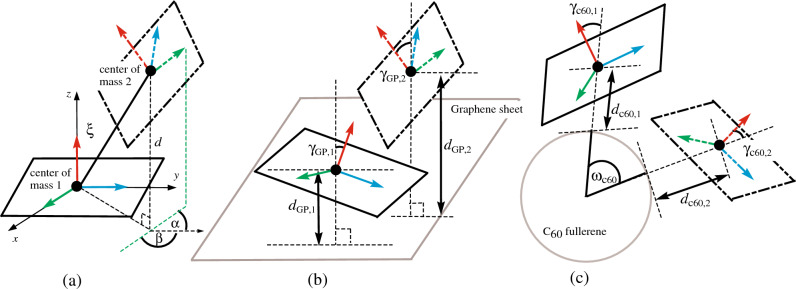
Schematic models for interaction between (**a**) two drug molecules, (**b**) two drug molecules and graphene and (**c**) two drug molecules and C_60_ fullerene.

Additionally, the obtained configurations mostly appear to be parallel structures, measured from the defined vectors. However, for precise atomic positions on the molecule, additional parameters are necessary as illustrated in Fig. 3. Regarding the interaction between two drug molecules, as shown in Fig. 3a, $\xi$ (Å) represents the distance between the centers of masses of the two molecules, and *d* (Å) is considered the shortest distance between two parallel planes. Furthermore, $\alpha$ and $\beta$ (degrees) denote the incline angle and the rotational angle between the two molecules. In Supplementary Material S1, various alignments of two drug molecules with different values of $\alpha$ and $\beta$ are demonstrated.

In conjunction with the parameters outlined for the interaction between two drug molecules, Fig. 3b introduces additional parameters for the interaction between a drug and a flat graphene sheet. In this context, the distance from the center of mass of a drug molecule to the graphene is denoted as $d_{GP}$ (Å). In the case of multiple drug molecules, each specific molecule is assigned an additional index. The tilted angle $\gamma _{GP}$ (degrees) measures the alignment of the drug carbon plane being parallel to the graphene with the normal vector. In Fig. 3c for the C_60_ fullerene, the shortest distance between the C_60_ surface and the drug center of mass is represented by $d_{C60}$ (Å). Similar to the graphene case, $\gamma _{C60}$ denotes the tilted angle between the normal vector of the drug molecule and the C_60_. Additionally, $\omega _{C60}$ (degrees) illustrates the angular separation between two drugs on the surface of the C_60_.

Given the different drug types and nanocarriers in our experiments, we adopt a nomenclature for each system

$${\text{X}-}N{\text{Y}-\text{A},}$$

where X represents the nanocarrier which can be either GP or C60 to represent the graphene sheet or the C_60_ fullerene, respectively, and the nomenclature without X signifies the interaction between drug molecules. Here, *N* denotes a number of drug molecules and Y specifies the drug type, FU for fluorouracil, PF for proflavine, or MB for methylene blue. Further, letter A defines different configuration of the system, and if there is only one possible stable configuration, A will be omitted. For example, 3FU-B denotes configuration B for the interaction energy between three molecules of fluorouracil, GP-2MB indicates the system of graphene interacting with two methylene blues, and C60-1PF-A represents configuration A of the system of fullerene interacting with one proflavine molecule.

### Interaction energy of two identical drugs

**Figure 4:**
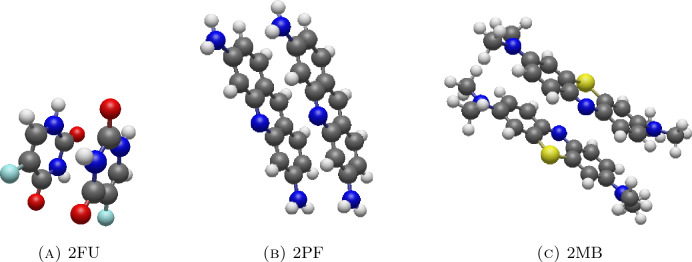
Equilibrium configurations for interaction between two molecules of (**A**) fluorouracils, (**B**) proflavines and (**C**) methylene blues.

We compute the non-bonded interaction energy between two drug molecules by discretely summing the interactions of each pair of atoms from the two molecules. Table 1 presents the values of $\alpha$ and $\beta$, indicating a parallel alignment of the configurations. The system of 2FU suggests an orthogonally parallel arrangement, 2PF displays a clearly co-parallel structure, while 2MB exhibits an anti-parallel alignment (see Supplementary Material S1). Additionally, the stable configurations of the three systems seem to involve one molecule being directly above the other, as inferred from the values of *d* and $\xi$. The minimum energies for the systems of 2FU, 2PF, and 2MB are $-5.3235$, $-16.0845$, and $-21.8346$ kcal/mol, respectively, correlating with the molecular size. These results provide fundamental insights for our study, and their 3D molecular configurations are depicted in Fig. 4. It is worth noting that Avogadro2^28^ is employed for visualization purposes.

**Table 1:** Numerical results for interaction between two identical drugs.

Systems	*E* (kcal/mol)	*d* (Å)	$\xi$ (Å)	$\alpha$ (^∘^)	$\beta$ (^∘^)
2FU	$-5.3235$	3.447	3.448	179.059	82.127
2PF	$-16.0845$	3.454	3.521	1.007	7.149
2MB	$-21.8346$	3.497	3.647	179.867	12.264

### Interaction energy between graphene and drugs

**Figure 5:**
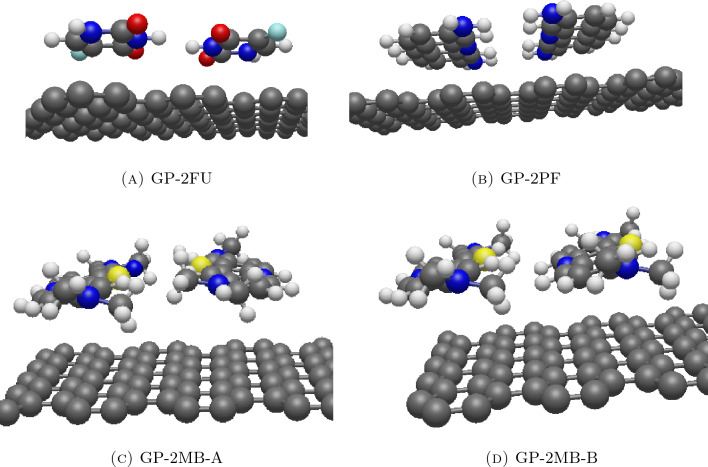
Equilibrium configurations for interaction between flat graphene sheet and two molecules of (**A**) fluorouracils, (**B**) proflavines, (**C**) methylene blues of configuration A and (**D**) methylene blues of configuration B.

We employ a hybrid discrete-continuous approach to examine the interaction energy between an infinite flat graphene sheet and an atom on the drug molecule. In this scenario, the flat graphene sheet serves as a nanocarrier for drug adsorption on its surface. Table 2 outlines the results of numerical experiments on molecular interactions with their 3D structures visualized in Fig. 5. As indicated by $\alpha$ and $\beta$, the configuration of two fluorouracils in GP-2FU demonstrates an anti-parallel arrangement (see Fig. 5a), distinct from the system of 2FU. In the GP-2PF system, two proflavine molecules exhibit an anti-parallel configuration (Fig. 5b), while the system with two methylene blue molecules displays two optimal configurations which are an anti-parallel alignment GP-2MB-A in Fig. 5c, and a co-parallel configuration GP-2MB-B in Fig. 5d.

Examining the values of $\gamma _{GP, 1}$ and $\gamma _{GP, 2}$ in Table 2, systems with one or two drug molecules show similar behavior with the carbon plane on the drug lying parallel to the graphene sheet. The systems of GP-1MB, GP-2MB-A, and GP-2MB-B may exhibit larger values of $\gamma _{GP, 1}$ and $\gamma _{GP, 2}$ due to their molecules do not being a perfectly flat plane. Additionally, the values of $d_{GP, 1}$ and $d_{GP, 2}$ for each drug type are equal, suggesting that the drug molecules are likely on the same layer. This is confirmed by the small value of *d*, the shortest distance between carbon planes of drugs. Furthermore, $\xi$ can be interpreted as the distance between two drugs where the distances in GP-2FU, GP-2PF, GP-2MB-A, and GP-2MB-B are 6.393, 6.839, 7.317, and 6.951 Å, respectively.

Moreover, we observe that the interaction energy between graphene sheet and a drug molecule is lower than that between two identical drug molecules (see Table 1). This leads us to the conclusion that the interaction energy between the graphene and the drug is more favorable than that between two drug molecules. Therefore, graphene sheet can be considered as a potential nanocarrier. Focusing on the minimum energy of GP-2FU, which is $-31.8970$ kcal/mol, and this may be viewed as the sum of energies from two systems of GP-1FU where we may deduce $2 \times (-15.3452) = -30.6904 > -31.8970 \,\, \text {kcal/mol}.$ Thus, the system of two fluorouracil molecules and graphene is more stable than the system of a single fluorouracil interacting with graphene. Similar conclusions can be drawn for the systems of GP-2PF, GP-2MB-A, and GP-2MB-B.

**Table 2:** Numerical results for interaction between graphene and drugs.

Drug	*E* (kcal/mol)	$d_{GP, 1}$ (Å)	$\gamma _{GP, 1}$ (^∘^)	$d_{GP, 2}$ (Å)	$\gamma _{GP, 2}$ (^∘^)	*d* (Å)	$\xi$ (Å)	$\alpha$ (^∘^)	$\beta$ (^∘^)
GP-1FU	$-15.3452$	3.348	0.667	–	–	–	–	–	–
GP-2FU	$-31.8970$	3.348	0.669	3.348	0.678	0.038	6.393	0.732	179.993
GP-1PF	$-34.8741$	3.362	0.816	–	–	–	–	–	–
GP-2PF	$-73.0596$	3.362	0.834	3.362	0.834	0.098	6.839	178.333	0.000
GP-1MB	$-39.8838$	3.684	11.428	–	–	–	–	–	–
GP-2MB-A	$-85.8370$	3.684	11.409	3.684	11.412	1.293	7.317	22.804	179.814
GP-2MB-B	$-85.5546$	3.681	12.392	3.691	11.781	1.496	6.951	0.617	0.051

**Table 3:** Numerical results for interaction between C_60_ and drugs.

Drug	*E* (kcal/mol)	$d_{C60, 1}$ (Å)	$\gamma _{C60, 1}$ (^∘^)	$d_{C60, 2}$ (Å)	$\gamma _{C60, 2}$ (^∘^)	*d* (Å)	$\xi$ (Å)	$\alpha$ (^∘^)	$\beta$ (^∘^)	$\omega _{C60}$ (^∘^)
C60-1FU	$-7.1061$	3.198	3.100	–	–	–	–	–	–	
C60-2FU-A	$-15.6584$	3.195	3.261	3.200	3.379	2.979	6.137	159.503	67.617	54.447
C60-2FU-B	$-15.6556$	3.192	3.260	3.198	3.524	2.748	5.945	15.726	169.870	52.631
C60-2FU-C	$-15.6508$	3.194	2.722	3.204	3.686	2.822	6.286	161.729	12.387	55.870
C60-1PF	$-12.3487$	3.116	0.852	–	–	–	–	–	–	
C60-2PF	$-29.4099$	3.103	0.790	6.568	3.706	3.444	3.509	1.012	7.769	3.898
C60-1MB	$-13.7382$	3.134	5.274	–	–	–	–	–	–	
C60-2MB-A	$-36.6135$	3.113	5.481	6.579	3.126	3.485	3.640	179.978	12.350	7.813
C60-2MB-B	$-36.5603$	3.114	4.697	6.679	9.368	3.506	3.598	1.545	18.013	3.427

### Interaction energy between C_60_ and drugs

Here, C_60_ fullerene is assumed to be a perfect sphere, the interaction energy between a sphere and a point is utilized. Then the discrete interaction energy from each atom on the drug is evaluated using the discrete summation to determine the adsorption energy of the drug on the surface of the spherical carrier. An example of the objective energy function for the C60-1FU system is provided in Supplementary Material S1. The numerical results for the stable configurations are given in Table 3. For systems with a single drug molecule, fluorouracil, proflavine and methylene blue have distances from the C_60_ surface of 3.198, 3.116 and 3.134 Å, respectively. Their carbon planes lie parallel to the surface of the C_60_ as indicated by small values of $\gamma _{C60, 1}$ which is less than $10^{\circ }$. Additionally, C60-1MB exhibits the lowest minimum energy due to the larger number of atoms in the molecule.

**Figure 6:**
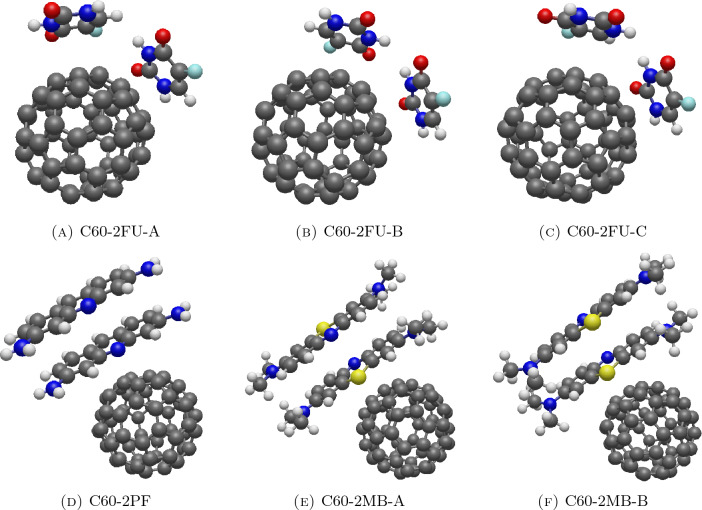
Equilibrium configurations for interaction between C_60_ and two molecules of (**A**) fluorouracils of configuration A, (**B**) fluorouracils of configuration B, (**C**) fluorouracils of configuration C, (**D**) proflavines, (**E**) methylene blues of configuration A and (**F**) methylene blues of configuration B.

In systems with two drug molecules, three equilibrium configurations are obtained for fluorouracils, one for proflavines, and two for methylene blues as illustrated in Fig. 6. For fluorouracils, $d_{C60, 1} \approx d_{C60, 2}$ and $\omega _{C60}$ is around $55^{\circ }$, suggesting a single-layer distribution on the C_60_ surface. Configurations C60-2FU-A, C60-2FU-B, and C60-2FU-C form different configurations which are orthogonally parallel (Fig. 6a), anti-parallel in rotation (Fig. 6b) and anti-parallel in flipping (Fig. 6c), respectively. Due to the curvature of the C_60_ surface, these configurations are not perfectly aligned as defined in Supplementary Material S1.

For C60-2PF, C60-2MB-A and C60-2MB-B, values of $\gamma _{C60, 1}, \gamma _{C60, 2}$, and $\omega _{C60}$ are small suggesting a stacked layer. This might be because the interacting surfaces of proflavine and methylene blue are relatively large compared to the smaller interacting surface of the C_60_, leading them to prefer a vertical stacking to maximize the interaction between atoms. Proflavines exhibit a vertically stacked structure in a co-parallel manner as depicted in Fig. 6d, while methylene blues display two possible equilibrium configurations, one is an anti-parallel and the other is a co-parallel structure as depicted in Fig. 6e,f, respectively. This is also confirmed by the values of $\alpha$ and $\beta$.

Regarding minimum energy, once again, the energy from the system containing two drug molecules interacting with the C_60_ is lower than the sum of energies from two systems containing a single drug molecule interacting with the C_60_. Therefore, increasing the number of drug molecules enhances the system’s stability.

### Interaction energy between C_60_ and various numbers of fluorouracils

Due to the spread of fluorouracils on the C_60_ surface, we further explore the adsorption of three to seven fluorouracil molecules. The energies for each system are determined as $-25.0817$, $-34.7354$, $-43.5168$, $-53.0952$, and $-63.2864$ kcal/mol, respectively. It is evident that the energy decreases by 9 to 10 kcal/mol as the number of fluorouracils increases. The angular separation between the *m*th and *n*th fluorouracils, denoted as $\omega _{mn}$ (degrees), is defined, and the numerical results are presented in Table 4. Note that the values are averaged from the three configurations of C60-2FU given in Table 3 for the case of two fluorouracils in Table 4.

**Figure 7:**
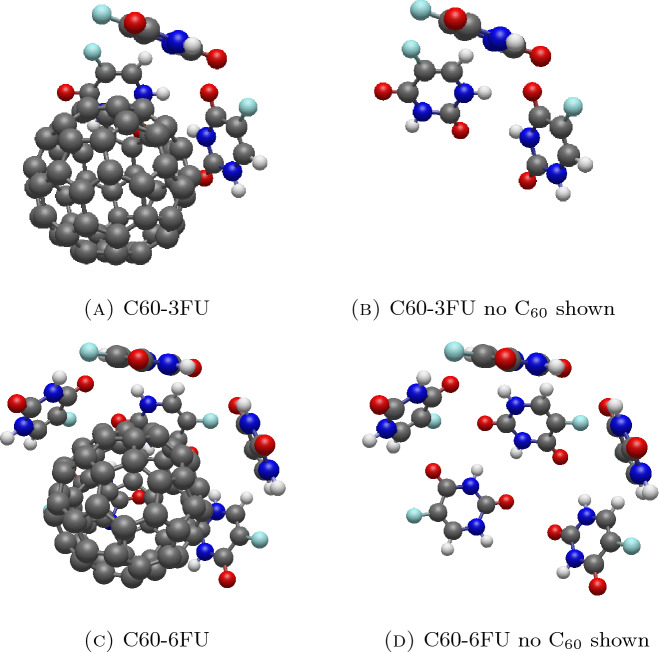
Equilibrium configurations for interaction between C_60_ and (**A,B**) three fluorouracils and (**C,D**) six fluorouracils.

In C60-3FU, fluorouracils align to form an equilateral triangle with a distance between their centers of masses, $\xi$, ranging from approximately 6.322–6.845 Å. As the number of fluorouracil molecules increases, they rearrange themselves to maintain an equilateral triangle on the surface of C_60_ while preserving the distance $d_{C60}$ and the interacting angle $\gamma _{C60}$. The systems of C60-3FU and C60-6FU are depicted in Fig. 7, and for a clearer view of the fluorouracil alignment, Fig. 7b,d illustrate the systems without the C_60_ molecule.

The alignments of three and six fluorouracil molecules on the graphene surface are also investigated to clarify the triangular alignment. For GP-3FU and GP-6FU, all fluorouracils maintain an average distance from the graphene of approximately 3.344 Å, similar to GP-1FU and GP-2FU. Additionally, the fluorouracil molecules align in an equilateral triangle, with distances between the centers of masses $\xi$ corresponding to the sides of the triangles measuring around 6.900–7.581 Å, which are larger than the value of $\xi$ for GP-2FU given in Table 2.

**Table 4:** Numerical results for interaction between C_60_ and various number of fluorouracil molecules, where $\omega _{mn}$ (^∘^) denoted angular separation between $m^{th}$ and $n^{th}$ fluorouracils.

No. of FU	2	3	4	5	6	7
*E* (kcal/mol)	$-15.6550$	$-25.0817$	$-34.7354$	$-43.5168$	$-53.0952$	$-63.2864$
$\omega _{12}$	54.316	56.235	57.316	56.401	56.993	54.494
$\omega _{13}$	–	61.390	102.531	58.371	105.036	103.549
$\omega _{23}$	–	56.949	57.050	58.086	119.520	56.257
$\omega _{14}$	–	–	58.536	62.218	60.009	58.259
$\omega _{24}$	–	–	60.547	65.360	110.381	61.872
$\omega _{34}$	–	–	56.260	113.183	55.110	109.905
$\omega _{15}$	–	–	–	56.160	107.864	102.898
$\omega _{25}$	–	–	–	104.099	72.601	58.678
$\omega _{35}$	–	–	–	107.422	58.740	62.271
$\omega _{45}$	–	–	–	55.529	102.530	59.500
$\omega _{16}$	–	–	–	–	56.785	157.118
$\omega _{26}$	–	–	–	–	63.021	102.675
$\omega _{36}$	–	–	–	–	60.645	57.886
$\omega _{46}$	–	–	–	–	59.735	112.450
$\omega _{56}$	–	–	–	–	55.582	57.916
$\omega _{17}$	–	–	–	–	–	58.406
$\omega _{27}$	–	–	–	–	–	57.416
$\omega _{37}$	–	–	–	–	–	59.950
$\omega _{47}$	–	–	–	–	–	109.126
$\omega _{57}$	–	–	–	–	–	109.650
$\omega _{67}$	–	–	–	–	–	113.214

## Discussion

Overall, graphene sheet appears to be a better nanocarrier than spherical C_60_ for fluorouracils, proflavines, and methylene blues. The results clearly indicate that the nanocarrier configuration adeptly interacts with the atoms on the drug’s carbon planes. This finding aligns with Neal et al.^9^ who employ the DFT to optimize the non-covalent interaction between C_60_ and DOX, epirubicin, and mitomycin, and report that the drug rings are prefer to be parallel to the five or six-membered rings of C_60_.

To further validate the advantage of our mathematical approach and optimization method in reducing computational time, we compare our results with previous works. Yaraghi et al.^8^ use the DFT to obtain an energy of approximately $-1.9140$ kcal/mol for the interaction between fluorouracil and a silicon graphene sheet. This value appears relatively low compared to our result from the system of GP-1FU. This difference may be attributed to other non-van der Waals energy involved in the interaction energy, as well as the position and configuration of fluorouracil with silicon graphene sheet in their study.

As demonstrated by Sun et al.^7^, molecular dynamics simulations are utilized to calculate proflavine docking scores into ChT, with and without pristine graphene, which can be considered as a proflavine molecule encapsulated inside a sphere. In their study, the docking scores range from $-17.74$ to $-23.77$ kcal/mol falling between the interaction energies of C60-1PF (see Table 3) and GP-1PF (see Table 2). Thus, proflavine exhibits moderate interactions with ChT whether graphene is present or not^7^. These interactions, however, are not as robust as those observed in GP-1PF where a parallel orientation is evident. Conversely, proflavine interactions with the surface of C_60_ are weaker likely due to the relative size between proflavine and the curvature of the C_60_ surface.

Last but not least, we draw a comparison between our study and the investigation conducted by Evstigneev et al.^10^ on C_60_ complexation in an aqueous solution with DOX, methylene blue, and proflavine using atomic force microscopy. They report the van der Waals interactions in a non-aqueous environment of $-13.4$ and $-20.4$ kcal/mol for proflavine and methylene blue systems, respectively. These values closely align with our energy results for C60-1PF, with a 6–7 kcal/mol difference observed for C60-1MB as presented in Table 3. Furthermore, they calculate the minimum distance between the carbons of the fullerene and the atoms of the ligand chromophores as 3.21 Å for the proflavine and 3.29 Å for the methylene blue. These findings are consistent with our study, where we compare these distances to our parameter $d_{C60,1}$ which are 3.116 and 3.134 Å, respectively. However, it is noteworthy that in our study, only fluorouracil is observed to distribute on the surface of C_60_, while proflavine and methylene blue tend to stack in multiple layers as the number of molecules increases, differing from their prediction^10^.

## Summary

This research aims to explore how combining explicit formulas with the U-NSGA-III algorithm can help identify stable configurations of drug molecules interacting with nanocarriers. We use fluorouracil, proflavine, and methylene blue as examples of chemotherapy drugs, and graphene sheets and C_60_ fullerene as nanocarriers. To model irregularly shaped drug molecules, we use a hybrid discrete-continuous approximation based on the Lennard-Jones potential function for non-bonded interactions.

The study initially derives analytical expressions for the interaction energy between an atom and an infinite flat plane, as well as between an atom and a spherical surface. These expressions are instrumental in evaluating the interactions between drug atoms and graphene or C_60_ surfaces. Subsequently, the U-NSGA-III algorithm from the pymoo library is applied to optimize the energy with respect to the relative positions of molecules.

The outcomes of our approach facilitate the straightforward determination of atomic positions for both drugs and nanocarriers at equilibrium. The observed equilibrium energy between two drug molecules is surpassed by the energy between two drugs and the carrier, suggesting a proclivity for drug attachment to the nanocarrier surface. The stability of the system is influenced significantly by the carbon planes on the drug molecules with both drug and carrier favoring maximal surface contact.

Our findings reveal that, due to the expansive flat surface of graphene, all three drug types spread on its surface, whereas only fluorouracils adhere to the C_60_ surface. Consequently, C_60_ fullerene emerges as a promising nanocarrier for fluorouracil, while its surface proves insufficient for proflavine and methylene blue. Moreover, an increased number of drug molecules correlates with lower equilibrium energy.

These results, derived from fundamental mathematical principles and heuristic procedures, align closely with outcomes obtained through computationally intensive methods. Thus, this theoretical exploration serves as a foundational step in the development of a nanocarrier for drug delivery systems.

### Supplementary Information

Supplementary Information.

## Data Availability

The datasets used and/or analysed during the current study available from the corresponding author on reasonable request.
