# Supplementary material for: Mathematical modeling and optimization technique of anticancer antibiotic adsorption onto carbon nanocarriers
Source: Sci Rep. 2024 May 25;14:11988. doi: 10.1038/s41598-024-62483-4 (PMC11127958; doi:10.1038/s41598-024-62483-4)
Supplement: Supplementary file 1 — Supplementary Information. [file 41598_2024_62483_MOESM1_ESM.pdf]

Supplementary Material - Mathematical modeling and optimization technique of anticancer antibiotic adsorption onto carbon nanocarriers

May 8, 2024

1 Continuous and discrete approximations

We tested the mathematical expressions derived from the continuous approximation of two molecules and the hybrid discrete-continuous approximation. In the continuous approach, the equilibrium interaction energy between two spherical C_{60} fullerenes is -9.88243 kcal/mol with the distance between their centers of 10.02325 Å. The equilibrium interaction energy between a spherical C_{60} fullerene and an infinite flat graphene sheet is -29.19610 kcal/mol, and the distance from the center of the fullerene to the graphene is 6.49861 Å.

In hybrid discrete-continuous approximation, the graphene is assumed to be a continuous plane whereas the fullerene is considered as a discrete structure. The optimal interaction energy between the C_{60} fullerene as a discrete molecule and the infinite flat graphene sheet is -30.67018 kcal/mol, and a distance from the center of the fullerene to the graphene is 6.37027 Å. Assuming one fullerene molecule to be a

perfect spherical structure interacting with another discrete fullerene structure yields a minimum energy of -11.17408 kcal/mol and the distance between their center of 9.59308 Å. Furthermore, for the discrete summation between two C_{60} fullerenes, we obtain the energy of -12.06251 kcal/mol with the distance between their centers of 8.76983 Å.

When comparing the interaction between two C_{60} fullerenes, each system exhibits an energy difference of 1 kcal/mol, which would decrease with a higher number of atoms in the system. The energies obtained from the two models for the interaction between C_{60} and graphene are in good agreement, and the separation distances are approximately equal.

2 Energy function for optimization

In this appendix, energy equations obtained from the hybrid approach for the system involving fluorouracil molecule $C_4H_3FN_2O_2$ are given. For the interaction with the flat graphene sheet, we employ the interaction energy between a point and the infinite plane given in (3) and sum over 12 atoms of the fluorouracil, then the total interaction energy between one fluorouracil and graphene is given by

$$\begin{aligned} E_p^{tot} &= \eta_p \pi \Bigg[\sum_{i_C=1}^{4} \left(-\frac{A_{CC}}{2\delta_{i_C}^4} + \frac{B_{CC}}{5\delta_{i_C}^{10}} \right) + \sum_{i_H=1}^{3} \left(-\frac{A_{CH}}{2\delta_{i_H}^4} + \frac{B_{CH}}{5\delta_{i_H}^{10}} \right) + \left(-\frac{A_{CF}}{2\delta_{i_F}^4} + \frac{B_{CF}}{5\delta_{i_F}^{10}} \right) \\ &\quad + \sum_{i_N=1}^{2} \left(-\frac{A_{CN}}{2\delta_{i_N}^4} + \frac{B_{CN}}{5\delta_{i_N}^{10}} \right) + \sum_{i_O=1}^{2} \left(-\frac{A_{CO}}{2\delta_{i_O}^4} + \frac{B_{CO}}{5\delta_{i_O}^{10}} \right) \Bigg], \end{aligned} \tag{2.1}$$

where δ represents the distance between an atom and the graphene surface which is the absolute of z-component of each atom in the optimization process, η_p is the mean atomic surface density of the graphene, and A_{MN} and B_{MN} denote the attractive and repulsive Lennard-Jones constants, respectively, utilizing the mixing rule of two

atomic species M and N.

For the system of two fluorouracil molecules, we use the energy equation (2.1) for each of the fluorouracil molecule and combine with the energy contribution using discrete approach for the interaction between two molecules of fluorouracil.

In the system of C_{60}, we employ the total interaction energy between a surface of a sphere and a single atom given in (5) to determine the interaction energy between a fluorouracil molecule and a C_{60} fullerene which is given by

$$\begin{aligned} E_s^{tot} &= \sum_{i_C=1}^{4} E_{sp}(\delta_{i_C}, A_{CC}, B_{CC}) + \sum_{i_H=1}^{3} E_{sp}(\delta_{i_H}, A_{CH}, B_{CH}) + E_{sp}(\delta_{i_F}, A_{CF}, B_{CF}) \\ &\quad + \sum_{i_N=1}^{2} E_{sp}(\delta_{i_N}, A_{CN}, B_{CN}) + \sum_{i_O=1}^{2} E_{sp}(\delta_{i_O}, A_{CO}, B_{CO}). \end{aligned} \quad (2.2)$$

Again the energy of two fluorouracil molecules intercting with the C_{60}, the interaction between two fluorouracils is calculated using the discrete summation and equation (2.2) is evaluated for each of the fluorouracil for the total energy of the system.

In the optimization process, we minimize the total energy equation in order to find the stable configuration where all atomic positions are obtained and they are available upon request.

3 Alignment of drugs based on incline and rotational angles

This appendix shows possible alignments of two drug molecules using the incline angle α and the rotational angle β. There are three configurations which defined as (i) co-parallel, (ii) anti-parallel and (iii) orthogonally parallel. Due to a symmetry of

proflavine molecule, they have less possible alignments and the schematic model for their arrangements is shown in Fig. 3.1. In the cases of fluorouracil and methylene blue, their possible arrangements are illustrated in Fig. 3.2.

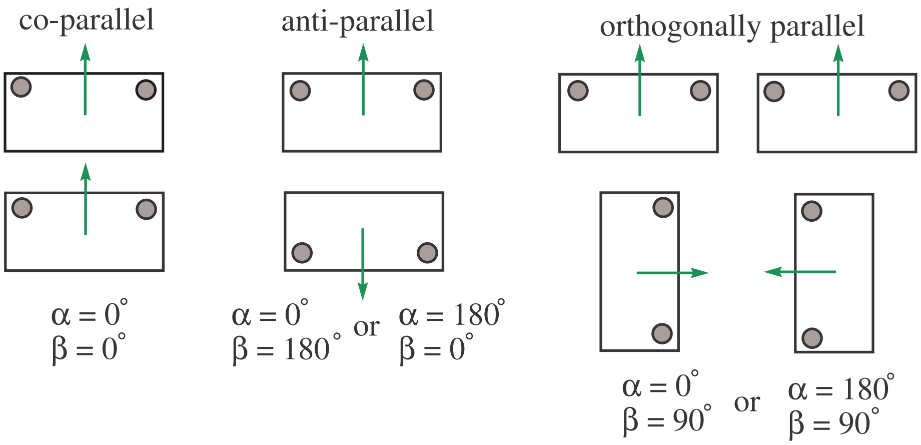

Figure 3.1: Schematic models for alignments of proflavine.

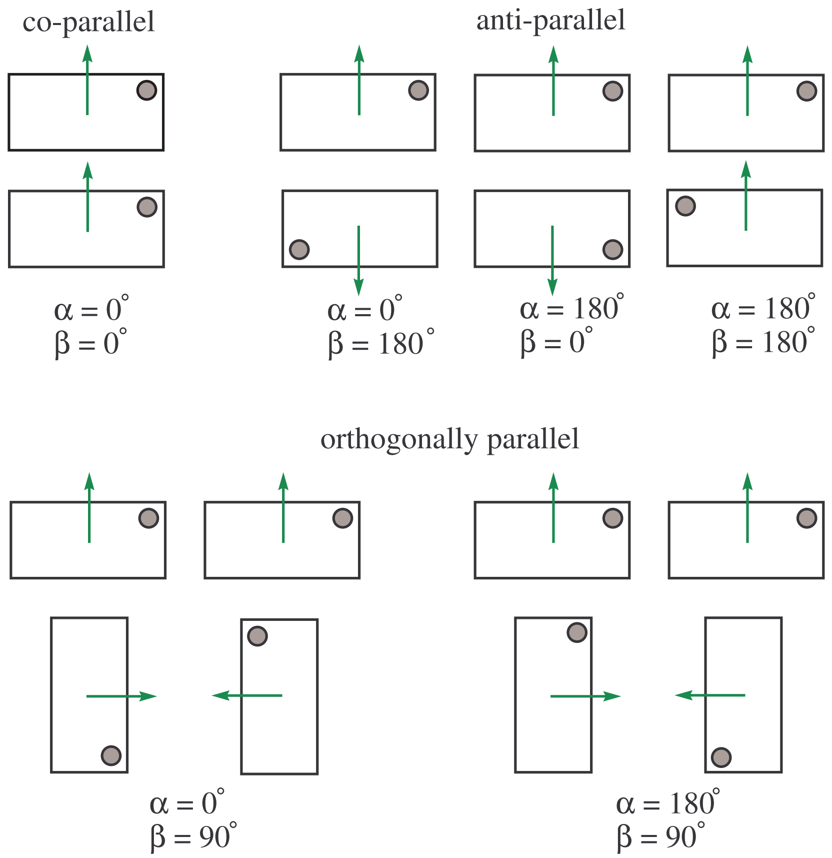

Figure 3.2: Schematic models for alignments of fluorouracil and methylene blue.
